# Targeted inhibition of osteoclastogenesis reveals the pathogenesis and therapeutics of bone loss under sympathetic neurostress

**DOI:** 10.1038/s41368-022-00193-1

**Published:** 2022-08-01

**Authors:** Bingdong Sui, Jin Liu, Chenxi Zheng, Lei Dang, Ji Chen, Yuan Cao, Kaichao Zhang, Lu Liu, Minyan Dang, Liqiang Zhang, Nan Chen, Tao He, Kun Xuan, Fang Jin, Ge Zhang, Yan Jin, Chenghu Hu

**Affiliations:** 1 State Key Laboratory of Military Stomatology and National Clinical Research Center for Oral Diseases and Shaanxi International Joint Research Center for Oral Diseases, Center for Tissue Engineering, School of Stomatology, The Fourth Military Medical University, Xi’an, China; 2 Law Sau Fai Institute for Advancing Translational Medicine in Bone and Joint Diseases, School of Chinese Medicine, Hong Kong Baptist University, Hong Kong SAR, China; 3 Xi’an Key Laboratory of Stem Cell and Regenerative Medicine, Institute of Medical Research, Northwestern Polytechnical University, Xi’an, China; 4 Xi’an Institute of Tissue Engineering and Regenerative Medicine, Xi’an, China; 5 Department of Biochemistry and Molecular Biology, School of Basic Medical Sciences, Xi’an Jiaotong University, Xi’an, China

**Keywords:** Antagomir and RNA sponge, Mechanisms of disease, Bone, Osteoporosis, Molecular medicine

## Abstract

Sympathetic cues via the adrenergic signaling critically regulate bone homeostasis and contribute to neurostress-induced bone loss, but the mechanisms and therapeutics remain incompletely elucidated. Here, we reveal an osteoclastogenesis-centered functionally important osteopenic pathogenesis under sympatho-adrenergic activation with characterized microRNA response and efficient therapeutics. We discovered that osteoclastic miR-21 was tightly regulated by sympatho-adrenergic cues downstream the β2-adrenergic receptor (β_2_AR) signaling, critically modulated osteoclastogenesis in vivo by inhibiting programmed cell death 4 (Pdcd4), and mediated detrimental effects of both isoproterenol (ISO) and chronic variable stress (CVS) on bone. Intriguingly, without affecting osteoblastic bone formation, bone protection against ISO and CVS was sufficiently achieved by a (D-Asp_8_)-lipid nanoparticle-mediated targeted inhibition of osteoclastic miR-21 or by clinically relevant drugs to suppress osteoclastogenesis. Collectively, these results unravel a previously underdetermined molecular and functional paradigm that osteoclastogenesis crucially contributes to sympatho-adrenergic regulation of bone and establish multiple targeted therapeutic strategies to counteract osteopenias under stresses.

## Introduction

The last two decades have witnessed the discovery that the brain and the skeleton are interconnected in physiology and pathology, in which a series of released factors mediate the mutual regulatory effects.^1–4^ Particularly, the central regulation of bone remodeling origins from the hypothalamus via the sympathetic nerve fibers to function on adrenergic receptors (ARs), which is implicated in psychological stress and profoundly disrupts bone homeostasis in human.^3–6^ Current understanding of bone alterations in responses to sympatho-adrenergic stimuli is focused on osteoblasts with characterized expression of βARs and fine-tuning molecular cascades, which primarily lead to impairment of bone formation underlying the development of osteopenia.^4,7–10^ However, together with the differential and controversial effects of βARs on bone, the use of β-blockers has yet achieved consistent clinical benefits on bone, which implies that the pathogenesis of sympathetic osteopenia has not been fully dissected.^11–16^ Therefore, the mechanistic research remains as an unmet need and will shed light on feasible therapeutic strategies for bone loss under the sympathetic neurostress.

Osteoclasts play a crucial role being a physiological regulator of skeletal homeostasis and the therapeutic target for bone pathologies have long been recognized and have continued to be revealed by recent discoveries.^17–20^ Nevertheless, under the context of sympatho-adrenergic regulation, mechanistic understanding and therapeutic manipulation promise of osteoclastogenesis are underdetermined, with osteoblastic paracrine factors currently known to be responsible for coupling osteoclast formation.^7,9,21,22^ To address the mechanistic and therapeutic research needs, we have previously focused on investigating the post-transcriptional regulator microRNAs, and we found miR-21 to be a candidate to promote osteoclastogenesis in vivo and a biomarker for human osteoporosis.^22–24^ We have also established an osteoclast-selective delivery method carrying an agomir/antagomir of a specific microRNA by conjugating D-Asp_8_ (an aspartate octapeptide) with lipid nanoparticles.^18,25–27^ In fact, the nucleic acid therapeutics are a growing field for the long-lasting or even curative effects, the clinical translation of which depends on delivery technologies including the lipid nanoparticles.^28–30^ Osteoclast-targeted inhibition of miR-21 is thus feasible and would be of value to examine the skeletal pathogenesis and therapeutics under sympatho-adrenergic cues.

Notably, in a recent study, we have shown that sympathetic regulation of the skeleton is mediated through osteoblast-osteoclast miR-21 communication via exosomes,^22^ but the core concept underlying sympathetic regulation of bone remains consistent with previous reports, i.e. osteoblastic response represents as crucial and dominant. In this further research, enlightened by the potential function of miR-21 in dictating osteoclastogenesis, we aim to investigate whether osteoclastic miR-21 can be therapeutically targeted for counteracting sympatho-adrenergic activation-induced osteopenia and whether osteoclastogenesis serves as a critical pathogenesis of bone loss under the sympathetic neurostress, which has not yet been identified. We discovered that osteoclastic miR-21 was regulated by sympatho-adrenergic cues and that osteoclast-specific manipulation of miR-21 was achieved by applying the (D-Asp_8_)-lipid nanoparticle-based nucleic acid delivery system. Taking advantage of this targeted technique, we confirmed that osteoclastic miR-21 was critical to modulate osteoclastogenesis and mediate the detrimental effects of the β_1/2_AR agonist isoproterenol (ISO) on bone homeostasis, as well as the chronic variable stress (CVS), which was due to osteoclastic miR-21 responding to receptor activator of nuclear factor-kappa B ligand (RANKL) with reducing programmed cell death 4 (Pdcd4). Further beyond molecular targets, ISO- and CVS-induced osteoclastogenesis can be therapeutically suppressed by clinically relevant drugs, RANK-Fc and clodronate, which were sufficient to ameliorate ISO- and CVS-induced osteopenias despite the not rescued osteoblastic bone formation rates. These findings together reveal an underdetermined pathogenic paradigm which osteoclastogenesis critically contributes to sympatho-adrenergic activation-induced osteopenia and establish targeted therapeutics to protect bone mass despite the psychological stress.

## Results

### Sympatho-adrenergic regulation of bone involves osteoclastic MiR-21 response

**Fig. 1:**
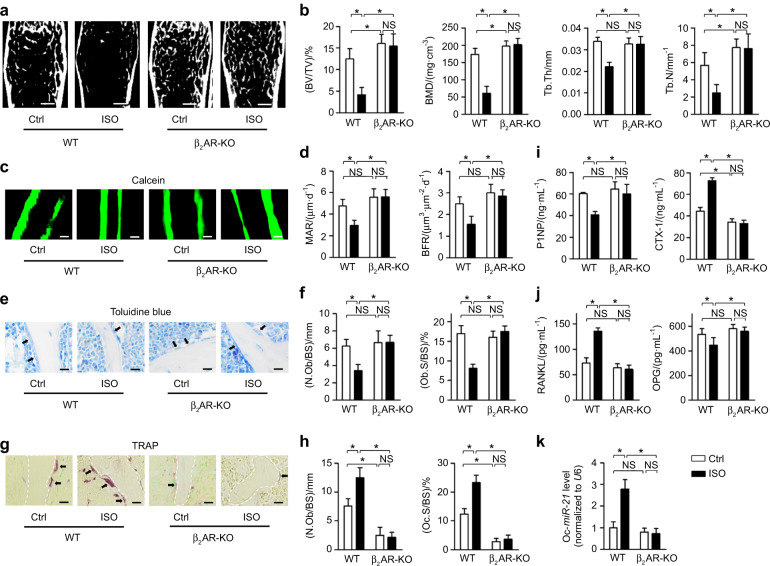
Sympatho-adrenergic regulation of bone involves osteoclastic miR-21 response. **a**, **b** Micro-CT analysis of femoral bone mass and trabecular parameters. Bars: 500 μm. **c**, **d** Calcein labeling of mineralized femoral surface and quantification of bone formation rates. Bars: 50 μm. **e**, **f** Toluidine blue staining showing osteoblasts (black arrows indicated) and the quantification. Bars: 12.5 μm. **g**, **h** TRAP staining for osteoclasts (black arrows indicated) and the quantification. White dashed lines indicate borders of the trabecular bone. Bars: 12.5 μm. **i**, **j** ELISA analysis of serum bone remodeling markers. **k** MiR-21 expression levels in isolated osteoclasts (Oc). *n* = 6 per group (**a**–**j**) and *n* = 3 per group (**k**). WT or β_2_AR knock-out mice received the ISO challenge or PBS injection. Mean ± SD. *P < 0.05; NS not significant

We first deciphered the significance of sympathetic control of bone through the downstream β-AR signaling.^31^ As the β_3_AR was excluded from regulation of the skeleton,^13^ to reliably and optimally mimick the physiological sympathetic tone, we accordingly applied ISO for bone regulation.^4,10^ Expectedly, micro-CT examination and corresponding tranbecular quantification demonstrated that daily intermittent injection of ISO for 1 month in mice induced remarkable osteopenia (Fig. 1a, b). The ISO challenge also dramatically impaired the bone remodeling balance, which was characterized by a suppressed bone formation rate examined by calcein labeling (Fig. 1c, d), a diminished osteoblastic number and surface shown by toluidine blue staining (Fig. 1e, f), an elevated osteoclastic bone resorption evaluated by activity of tartrate resistant acid phosphatase (TRAP) (Fig. 1g, h), a reduced level of the serum bone formation marker procollagen 1 N-terminal peptide (P1NP) with elevated serum bone resorption marker cross-linked C-telopeptide of type 1 collagen (CTX-1) (Fig. 1i), and an increased serological concentration of RANKL with decreased osteoprotegerin (OPG) (Fig. 1j). Moreover, although that the relative importance and functional contributions of β_2_AR to the sympathetic control of bone remain controversial among the ARs,^7,9,13–16^ we discovered that mice deficient for only β_2_AR were protected from ISO-induced osteopenia and substantially preserved bone homeostasis under the ISO challenge (Fig. 1a–j). The potent skeletal protective effects of β_2_AR deficiency were additionally revealed in the estrogen deficient ovariectomy (OVX) mouse model^32^ (Fig. S1a–j). Previously, we have screened and found miR-21 as the most prominent sympatho-adrenergic tone-responsive microRNA in bone, and that miR-21 primarily regulated osteoclastogenesis in vivo.^22–24^ Here, upregulation of osteoclastic miR-21 was further confirmed after ISO injection, which, however, did not occur in the lack of β_2_AR (Fig. 1k). Taken together, the collected data indicated the involvement of osteoclastic miR-21 in the sympatho-adrenergic regulation of bone.

### Selective suppression of osteoclastic MiR-21 retards osteoclastogenesis and ameliorates osteopenia provoked by ISO

**Fig. 2:**
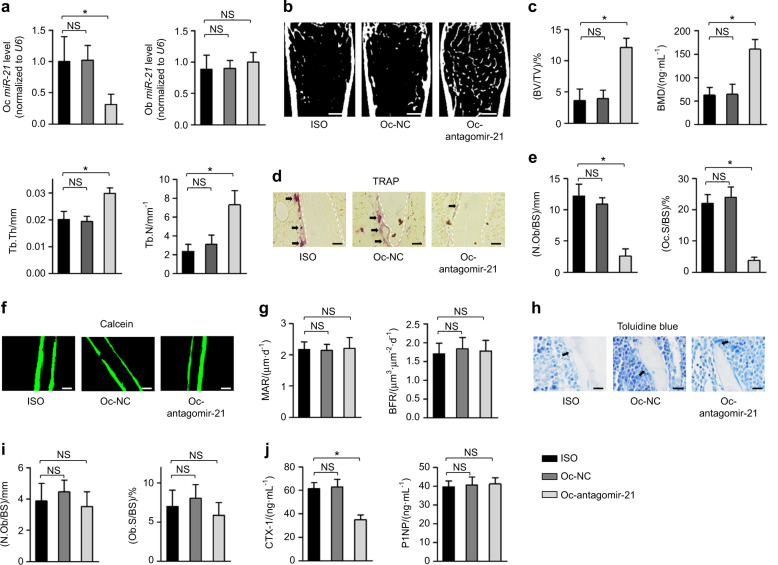
Selective suppression of osteoclastic miR-21 retards osteoclastogenesis and ameliorates osteopenia provoked by ISO. **a** MiR-21 expression levels in isolated osteoclasts (Oc) and osteoblasts (Ob). **b**, **c** Micro-CT analysis of femoral bone mass and trabecular parameters. Bars: 500 μm. **d**, **e** TRAP staining for osteoclasts (black arrows indicated) and the quantification. White dashed lines indicate borders of the trabecular bone. Bars: 12.5 μm. **f**, **g** Calcein labeling of mineralized femoral surface and quantification of bone formation rates. Bars: 50 μm. **h**, **i** Toluidine blue staining showing osteoblasts (black arrows indicated) and the quantification. Bars: 12.5 μm. **j** ELISA analysis of serum bone remodeling markers. *n* = 3 per group (**a**) and *N* = 5 per group (**b**–**j**). Osteoclast-targeted delivery of antagomir-21 (Oc-antagomir-21) or its negative control (Oc-NC) was performed under the ISO challenge. Mean ± SD. *P < 0.05; NS not significant

Next, we investigated whether inhibition of osteoclastic miR-21 provides a feasible strategy to protect bone mass against sympatho-adrenergic activation. In this regard, we applied (D-Asp_8_)-lipid nanoparticles to deliver microRNA modulators specifically to osteoclasts.^18,25^ As detected, effective antagomir-21 administration in vivo to osteoclasts was verified, with the specificity of this system confirmed by the unaffected osteoblastic miR-21 expression (Fig. 2a). Importantly, osteoclast-targeted miR-21 suppression alleviated bone loss and improved trabecular microarchitecture under ISO (Fig. 2b, c). As expected, the skeletal protection effects were attributed to suppressed osteoclastogenesis by targeted osteoclastic miR-21 inhibition (Fig. 2d, e), while the bone formation rates and osteoblastic parameters were not influenced and remained impaired, shown by bone histomorphometric analyses (Fig. 2f–i). Enzyme-linked immunosorbent assay (ELISA) analysis of serum bone resorption and bone formation markers confirmed the bone histomorphometric results (Fig. 2j). These findings indicated that targeted suppression of osteoclastic miR-21 retards osteoclastogenesis and ameliorates ISO-induced osteopenia.

To further identify the key role of osteoclastic miR-21 in mediating sympatho-adrenergic regulation of bone, with the help of the (D-Asp_8_)-lipid nanoparticle nucleic acid delivery system, we specifically delivered agomir-21 into osteoclasts, without targeting osteoblasts, of the miR-21-deficient mice as a “rescue” experiment (Fig. S2a). According to our previous reports, miR-21 deficiency affects the bone remodeling balance and preserves bone homeostasis against multiple extrinsic factors.^22–24^ In this study, we further revealed that with only osteoclastic expression of miR-21, osteopenia developed under the ISO challenge (Fig. S2b, c) due to stimulated osteoclastic bone resorption with remained bone formation or the osteoblastic parameters (Fig. S2d–j). For the potential mechanism of miR-21 regulating osteoclastogenesis, we referred to our previous findings of miR-21 targeting and inhibiting Pdcd4.^22–24,33^ Immunohistochemistry (IHC) analysis showed that Pdcd4-positive cells around the trabecular bone surfaces were reduced after ISO injections, whereas the specific delivery of antagomir-21 into osteoclasts rescued Pdcd4-positive cells despite ISO, representing in vivo evidence that Pdcd4 decreased in response to sympatho-adrenergic cues and functioned downstream of miR-21 (Fig. S3a, b). We also applied targeted inhibition of osteoclastic Pdcd4 expression in the miR-21-deficient mice (Fig. S3c), and found that specific delivery of small interfering RNAs (siRNAs) of Pdcd4 into osteoclasts led to bone loss under the ISO challenge despite lack of miR-21 (Fig. S3d, e). Again, recovered osteoclastic bone resorption rather than osteoblastogenesis (Fig. S3f–l) underlay the effects. These data suggested the pivotal role and the Pdcd4-based mechanism of osteoclastic miR-21 in mediating ISO-induced osteoclastogenesis and bone loss.

### RANKL blockade retards ISO-induced osteopenia through inhibiting osteoclastic MiR-21

**Fig. 3:**
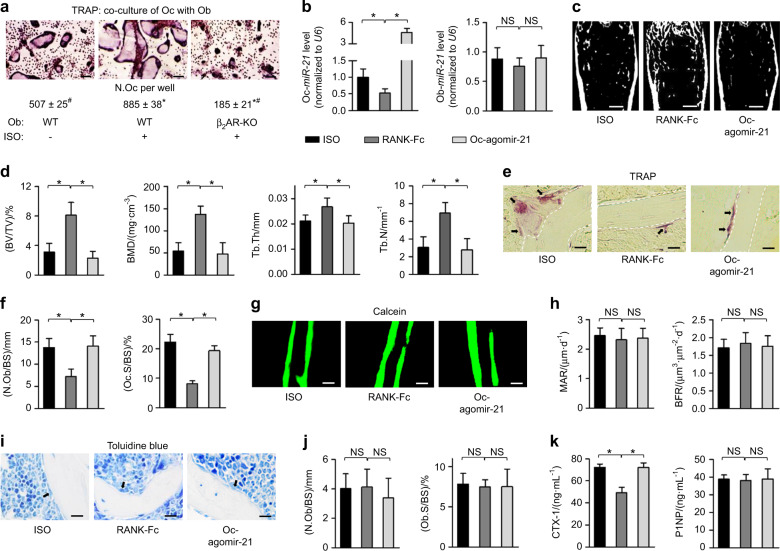
RANKL blockade retards ISO-induced osteopenia through inhibiting osteoclastic miR-21. **a** TRAP staining of osteoclasts (Oc) formed in direct co-culture with osteoblasts (Ob). Bars: 100 μm. *P < 0.05 compared to the WT-Ob without ISO group; ^#^*P* < 0.05 compared to the WT-Ob with ISO group. **b** MiR-21 expression levels in isolated osteoclasts (Oc) and osteoblasts (Ob). **c**, **d** Micro-CT analysis of femoral bone mass and trabecular parameters. Bars: 500 μm. **e**, **f** TRAP staining for osteoclasts (black arrows indicated) and the quantification. White dashed lines indicate borders of the trabecular bone. Bars: 12.5 μm. **g**, **h** Calcein labeling of mineralized femoral surface and quantification of bone formation rates. Bars: 50 μm. **i**, **j** Toluidine blue staining showing osteoblasts (black arrows indicated) and the quantification. Bars: 12.5 μm. **k** ELISA analysis of serum bone remodeling markers. *n* = 4 per group (**a**), *n* = 3 per group (**b**) and *n* = 5 per group (**c**–**k**). RANK-Fc injected with or without osteoclast-targeted delivery of agomir-21 (Oc-agomir-21) was performed under the ISO challenge. Mean ± SD. *P < 0.05; NS not significant

We and others have previously established that sympatho-adrenergic regulation of osteoclastic formation is dependent on osteoblastic factors, typically RANKL.^7,21,22^ Accordingly, when induced by adding equal levels of RANKL in culture, the osteoclastogenesis in the wild-type (WT) and β_2_AR conditions was comparable (Fig. S1k). Instead, when osteoclastogenesis was induced by co-culturing with osteoblasts without exogenous RANKL, ISO treatment was effective to promote osteoclast formation, which was blocked by lack of β_2_AR in osteoblasts (Fig. 3a). Further given that RANKL induces miR-21 expression in osteoclasts,^34^ we sought to evaluate whether suppression of RANKL was efficient to protect bone mass against sympatho-adrenergic activation and whether down-regulated osteoclastic miR-21 mediates the effects. To this end, we applied the RANKL antagonist RANK-Fc^35,36^ and specifically delivered agomir-21 into osteoclasts to potentially counteract RANK-Fc effects. We discovered that RANK-Fc administration significantly reduced the osteoclastic miR-21 level under the ISO challenge, which was rescued by osteoclast-specific agomir-21 delivery without off-target effects on osteoblasts (Fig. 3b). Importantly, RANK-Fc administration was effective to prevent bone loss against ISO, while osteoclast-specific delivery of agomir-21 blocked the therapeutic effects (Fig. 3c, d). Furthermore, with ISO injections, RANK-Fc protection of bone mass and miR-21 counteraction of RANK-Fc effects were due to regulation of osteoclastogenesis, rather than the modulation of bone formation rates or osteoblastogenesis (Fig. 3e–k). Taken together, the above findings indicated that RANKL blockade prevents ISO-induced osteopenia through inhibiting osteoclastic miR-21.

### Antiresorptive therapy by clodronate is sufficient to ameliorate bone loss against the ISO challenge

**Fig. 4:**
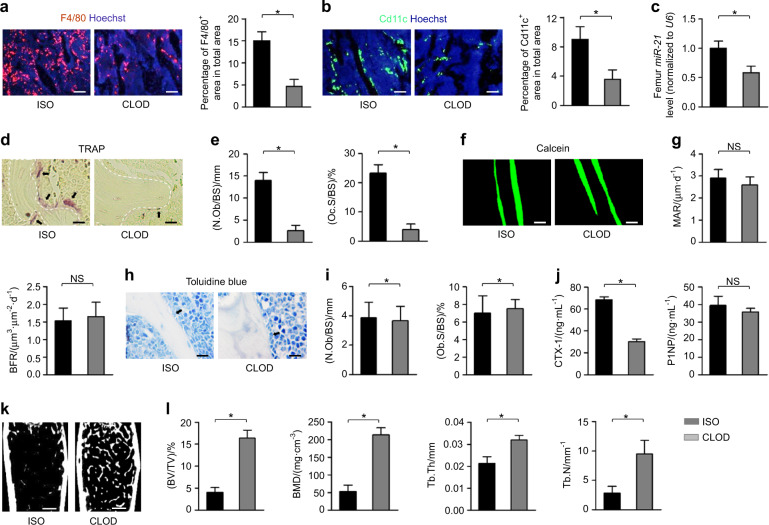
Antiresorptive therapy by clodronate is sufficient to ameliorate bone loss against the ISO challenge. **a**, **b** IF staining of the macrophage markers, F4/80 and Cd11c, in distal femora. Bars: 100 μm. **c** MiR-21 expression levels in femoral samples. **d**, **e** TRAP staining for osteoclasts (black arrows indicated) and the quantification. White dashed lines indicate borders of the trabecular bone. Bars: 12.5 μm. **f**, **g** Calcein labeling of mineralized femoral surface and quantification of bone formation rates. Bars: 50 μm. **h**, **i** Toluidine blue staining showing osteoblasts (black arrows indicated) and the quantification. Bars: 12.5 μm. **j** ELISA analysis of serum bone remodeling markers. **k**, **l** Micro-CT analysis of femoral bone mass and trabecular parameters. Bars: 500 μm. *n* = 6 per group (**a**, **c**–**l**) and *n* = 3 per group (**b**). Clodronate injection was performed under the ISO challenge. Mean ± SD. *P < 0.05; NS not significant

Although that current understanding of sympatho-adrenergic regulation of bone is focused on osteoblastic responses and effects,^4,7,8,10,22^ the above data inspire a functionally important osteopenic pathogenesis and efficient therapeutics under sympatho-adrenergic activation centered on osteoclastogenesis. Therefore, we then applied clodronate, a clinically approved bisphosphonate drug for selective depletion of macrophages and inhibition of osteoclastic activity,^37,38^ to test the efficacy of antiresorptive therapy on sympatho-adrenergic activation-induced osteopenia. As expected, clodronate injection remarkably reduced F4/80^+^ and Cd11c^+^ cell populations in bone despite ISO, which represented the effective ablation of general and proinflammatory macrophages (Fig. 4a, b).^39,40^ Intriguingly, clodronate application also suppressed the miR-21 expression in bone under the ISO challenge (Fig. 4c), together with dramatically inhibited osteoclastogenesis (Fig. 4d, e). Notably, the ISO-reduced osteoblastic bone formation rate was not restored by clodronate injection (Fig. 4f–i), as also supported by the analysis of serum bone remodeling markers (Fig. 4j), but ISO-induced osteopenia was substantially ameliorated (Fig. 4k, l). These results indicated an important finding that antiresorptive therapy by clodronate is sufficient to alleviate bone loss against sympatho-adrenergic activation.

### Targeted inhibition of osteoclastic MiR-21 counteracts osteopenia triggered by psychological stress

**Fig. 5:**
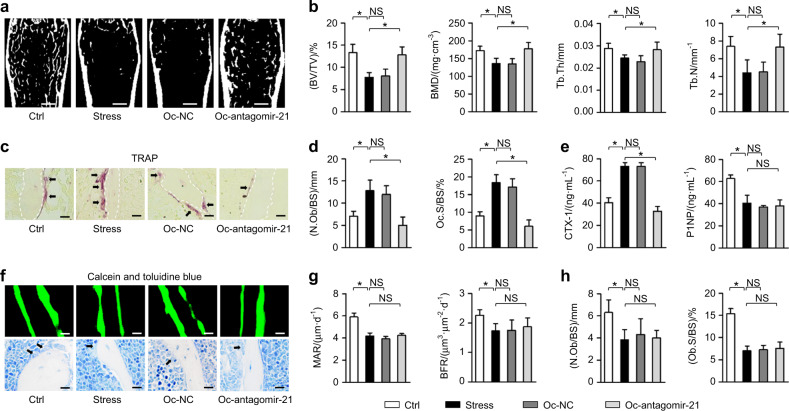
Targeted inhibition of osteoclastic miR-21 counteracts osteopenia triggered by psychological stress. **a**, **b** Micro-CT analysis of femoral bone mass and trabecular parameters. Bars: 500 μm. **c**, **d** TRAP staining for osteoclasts (black arrows indicated) and the quantification. White dashed lines indicate borders of the trabecular bone. Bars: 12.5 μm. **e** ELISA analysis of serum bone remodeling markers. **f**–**h** Calcein double labeling and toluidine blue images of mineralized femoral surface and osteoblasts (black arrows indicated) and the quantification. Bars: 50 μm (up) and 12.5 μm (down). *n* = 5 per group. Osteoclast-targeted delivery of antagomir-21 (Oc-antagomir-21) or its negative control (Oc-NC) was performed under the CVS modeling. Mean ± SD. *P < 0.05; NS not significant

Depression et al. psychological stresses is linked to notable bone loss and factures partially due to sympatho-adrenergic activation.^6^ We were further inspired to examine impacts of osteoclastic miR-21 and the more generalized osteoclastogenesis to stress-triggered bone loss and the potential to support therapeutics. Not surprisingly, exposure of mice to CVS resulted in loss of bone mass and reduced trabecular parameters (Fig. 5a, b), which were also characterized by impaired bone remodeling balance shown as increased osteoclastic bone resorption and decreased osteoblastic bone formation (Fig. 5c–h). Importantly, (D-Asp_8_)-lipid nanoparticle-facilitated administration of antagomir-21 selectively into osteoclasts retarded CVS-triggered osteopenia (Fig. 5a, b) through suppressing osteoclastogenesis, while the bone formation rate and osteoblastic parameters were not rescued (Fig. 5c–h). The above results demonstrated that osteoclastic miR-21 contributed to psychological stress-induced osteopenia which can be therapeutically targeted.

### Protection of bone mass under psychological stress is achieved by pharmacological suppression of osteoclastogenesis

**Fig. 6:**
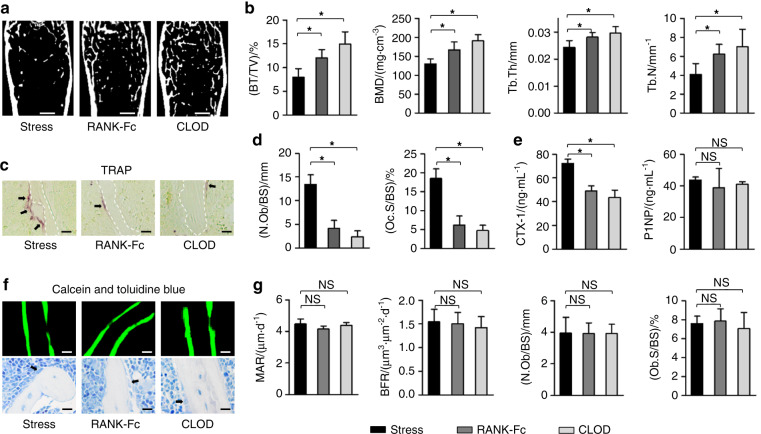
Protection of bone mass under psychological stress is achieved by pharmacological suppression of osteoclastogenesis. **a**, **b** Micro-CT analysis of femoral bone mass and trabecular parameters. Bars: 500 μm. **c**, **d** TRAP staining for osteoclasts (black arrows indicated) and the quantification. White dashed lines indicate borders of the trabecular bone. Bars: 12.5 μm. **e** ELISA analysis of serum bone remodeling markers. **f**–**h** Calcein double labeling and toluidine blue images of mineralized femoral surface and osteoblasts (black arrows indicated) and the quantification. Bars: 50 μm (up) and 12.5 μm (down). *n* = 6 per group. RANK-Fc or clodronate was injected under the CVS modeling. Mean ± SD. *P < 0.05; NS not significant

Finally, we applied the two clinically relevant chemical reagents, RANK-Fc and clodronate, for repressing bone resorption and treating CVS-induced osteopenia. Results showed that either RANK-Fc or clodronate administration was effective in counteracting bone loss under CVS, between which the effects of clodronate might be more prominent (Fig. 6a, b). Still, the therapeutic effects on CVS-induced osteopenia were attributed to reduced osteoclastogenesis, and the CVS-impaired osteoblastic bone formation was not rescued by RANK-Fc and clodronate (Fig. 6c–h). Collectively, these data indicated that efficient protection of bone mass under psychological stress is achieved by pharmacological suppression of osteoclastogenesis.

## Discussion

The bone stands as the major structural and an emerging endocrine organ to sustain the body homeostasis, yet the counteraction of extensive osteoporosis in various detrimental conditions represents an unmet current medical need.^41,42^ Anatomically, the bone forms an elaborate local milieu to various biological components, including the mesenchymal and hematopoietic lineage cells, the mineralized matrix, the vasculature and the innervation.^41,43^ In particular, the role of neural regulation of the skeleton has attracted plenty of recent research interest, which involves multiple neurologic factors secreted by sensory and autonomic nerve endings to influence the target bone cell function.^31^ Among them, the norepinephrine released by the sympathetic fibers has been revealed to participate in primary and secondary osteopenias in mice, and the sympatho-adrenergic control of bone has been crucially identified in a spectrum of physiological and pathological statuses of the skeleton, such as the hormonal actions, the emotional stress, the unloading and the tumorigenesis.^6,9,14,22,44^ In this study, we contribute to confirm that sympatho-adrenergic regulation is critical for bone homeostasis by applying ISO and β_2_AR deficiency in normal and OVX conditions, and we demonstrate that manipulation of adrenergic signaling cascades may serve as an efficient approach to treat osteoporosis. Of note, although we focused on β_2_AR, the dominant βAR subtype expressed on osteoblasts, effects of β_1_AR may play important roles in the OVX bone pathologies and upon mechanical stimulation.^15,16,22^ Future translational studies would be conducted based on our and others’ research so as to fulfill precise bone protection against the evolving neurostress in the modern society.

Previous mechanistic investigations on the sympathetic regulation of the skeleton have deciphered fine-tuning osteoblastic β_2_AR downstream signaling events, which involve the cyclic adenosine monophosphate (cAMP) response, the protein kinase A pathway, the osteopontin regulation, the transcription activation by cAMP response element-binding protein (Creb) as well as activating transcription factor 4 (ATF4), and responses of circadian clock genes, cyclins, microRNAs and RANKL.^7,8,10,21,22^ The predominant role of the molecules is to suppress osteoblastogenesis, while osteoclasts are shown to lack βARs and rely on osteoblast signals to initiate and maintain osteoclastogenesis under the sympatho-adrenergic context.^7,21,22^ However, as anabolic protection of bone by β-blockers might not be always effective,^11–16^ the detailed mechanism of sympathetic cue-triggered osteoclastic formation is worth to be further dissected thus providing novel targets for counteraction of bone loss under the neurostress. To the current knowledge, the differentiation of osteoclasts is centered on the tumor necrosis factor family cytokines (primarily RANKL) to stimulate the nuclear factor-kappa B signaling and transcription factor nuclear factor of activated T cells c1 (NFATc1)-mediated activation of osteoclastogenesis genes.^45^ Supported by targeted gene expression modulation, we hereby further show in vivo evidence that osteoclastic miR-21 responds to RANKL under the sympatho-adrenergic activation and targets Pdcd4 to drive osteoclastogenesis. In an in vitro study, miR-21 inhibition of Pdcd4 promoted the activator protein 1 complex containing c-Fos, which sustains an autoamplification loop to promote expression of miR-21 and NFATc1 thus enabling fast and robust induction of osteoclastogenesis.^34^ Therefore, osteoclastic miR-21 response to sympatho-adrenergic cues might not be specific. Importantly, even without rescue of bone formation, osteoclastic miR-21 inhibition and clinical-drug based antiresorptive therapy efficaciously counteract osteopenias under sympathetic neurostress, indicating the significance of osteoclastogenesis. Collectively, our research extends the current pathogenic and therapeutic landscape of sympathetic regulation of the skeleton.

MicroRNAs represent a post-transcriptional gene expression regulation mechanism which are increasingly recognized to modulate the skeletal health and sickness.^46,47^ Based on initial findings that miR-21 promotes both osteogenesis and osteoclastogenesis in vitro, we have previously reported that in mice lacking miR-21, increased physiological bone mass and protection against pathological bone loss are detected, with miR-21 regulation on osteoclastic bone resorption dominating.^23,34,48^ We have further documented that deficiency of miR-21 prevents the development of osteopenia under the ISO challenge, which is also attributed to the inhibition of osteoclastogenesis against the activation of the β_2_AR signaling cascades.^22^ Notably, miR-21 expression is significantly correlated with low BMD and osteoporotic fractures in human, indicating clinical relevance of this individual microRNA.^23,49^ Nevertheless, the efficacy of targeting in situ miR-21 for the osteoclastic regulation-based osteopenic therapy is not examined, and the in vivo mechanism of miR-21 modulating the osteoclastogenesis remains unveiled. In this study, we performed lipid nanoparticle-mediated targeted delivery of antagomirs, agomirs and siRNAs for osteoclast-specific manipulation, which holds translational promise for precise therapeutics against osteopenias and at the same time, have biological implications to confirm the molecular mechanism working in vivo. This strategy is of particular significance for targeting miR-21 as an antiresorptive therapy, given that global miR-21 knock-out exerts extensive effects in the organism which might not be beneficial.^50,51^ As the leading edge approach for translation,^28–30^ RNA therapeutics can be particularly conducted by liposomes modified with aptamers for targeted delivery of nucleic acids, which will prompt future studies to evaluate the potential in treating sympathetic bone loss, as we previously established.^52,53^

The sympathetic neurostress often occurs when an individual is exposed to the stressful stimuli in the modern life, which may eventually result in depressive disorders accompanied by reduced bone mass and increased disability.^5,54,55^ We hereby reveal that mice suffering from long-term unpredictable variable stresses develop sympathetic activation and bone loss. It is notable that chronic stresses also lead to a wide spectrum of pathologies, such as impaired immune function, diminished wound healing and hair growth arrest, in which either the sympathetic nervous system (SNS) or the hypothalamic-pituitary-adrenal (HPA) axis mediate the effects.^56–59^ Importantly, the sympathetic and CVS-induced osteopenias can both be effectively ameliorated by osteoclast-specific miR-21 inhibition, which provides at least one feasible approach to counteract the stress-related symptoms. Moreover, we show proof that RANK-Fc- and clodronate-mediated antiresorption also rescues the stress-triggered osteopenias, which holds immense promise for further translation. We assume that perhaps more efficient and extensive treatments to stress-induced pathologies across the organism are to intervene SNS and HPA axis per se, but the strategies may not be easy to establish and unintended outcomes might obtain because of the wide involvements of SNS and HPA axis in regulation of almost all tissues/organs in the body.^60–62^ Therefore, targeting the sympathetic downstream cascades serves as the feasible therapeutics and potential synergistic amelioration of multiple tissue/organ lesions under stress conditions remains to be investigated.

In summary, these results reveal a previously underdetermined molecular and functional paradigm that osteoclastogenesis crucially contributes to sympatho-adrenergic regulation of bone and establish multiple targeted therapeutics to protect the skeleton despite psychological stresses.

## Materials and methods

### Animals

WT mice (JAX no. 000664) and mice deficient for miR-21 (JAX no. 034191) or β_1/2_AR (JAX no. 003810) were purchased, crossed (WT with β_1/2_AR mice for the β_2_AR strain), maintained and used according to our previous studies.^22–24,33^

### Establishment of osteopenias

ISO injection, CVS modeling and OVX surgery with their controls were performed in mice following our previous studies.^10,22,32,57^

### Construction and application of the osteoclast-specific delivery system

(D-Asp_8_)-lipid nanoparticles were fabricated and utilized for osteoclast-selective nucleic acid delivery based on our previous protocol.^18,25^ (D-Asp_8_)-liposomes dissolved in 40 mg per 100 μl RNase-free saline were mixed with equal volume of RNase-free saline containing 200 μg agomir-21, antagomir-21, siPdcd4 or their respective negative control, which were all synthesized by RiboBio (Guangzhou, China). The mixture were incubated for 20 min at room temperature and injected via the caudal vein into mice at the nucleic acid dosage of 8 mg/kg every other week for 4 weeks.

### Pharmaceutical inhibition of osteoclastogenesis

RANK-Fc and clodronate were used as reported.^36,63^ Recombinant mouse RANK-Fc (692-RK, R&D Systems, USA) in saline was administered intraperitoneally at 10 mg·kg^−1^ three times per week for 1 month. Clodronate (233183, Sigma-Aldrich, USA) in saline was administered intraperitoneally at 50 mg·kg^−1^ three times per week for 1 month.

### Culture of mouse osteoclasts and co-culture with osteoblasts

Murine osteoclasts were cultured under 20 ng·mL^−1^ macrophage colony-stimulating factor (M-CSF, 315-02, Peprotech, USA) and 50 ng·mL^−1^ RANKL (315-11, Peprotech, USA) stimulation, and were co-cultured with murine osteoblasts under 20 ng·mL^−1^ M-CSF, 10 nM Vitamin D3 (HY-15398, MCE, China) and 1 μmol·L^−1^ prostaglandin E2 (PGE2) (P0409, Sigma-Aldrich, USA), as described.^22,23,32^ TRAP staining and ISO treatment was also applied accordingly.

### MiR-21 and mRNA expression analysis

Mouse femur tissues or cultured osteoclasts and osteoblasts were collected for RNA extraction, and reverse transcription and quantitative real-time polymerase chain reaction were performed as reported.^22,23^

### ELISA

The serum and media samples were isolated following our previous study.^22^ Concentrations of P1NP (NBP2-76466, Novus Biologicals, USA), CTX-1 (NBP2-69074, Novus Biologicals, USA), RANKL (MTR00, R&D Systems, USA) and OPG (MOP00, R&D Systems, USA) were determined using commercial kits.

### Micro-CT analysis

A desktop micro-CT system (eXplore Locus SP, GE Healthcare, USA) was utilized, and femora were scanned and quantified for trabecular parameters (BV/TV, bone volume over tissue volume; BMD, bone mineral density; Tb.Th, trabecular thickness; Tb.N, trabecular number), as we previously established.^22,23,32^

### Bone histomorphometric analysis

Double calcein labeling was performed and quantified for bone formation (MAR, mineral apposition rate; BFR, bone formation rate) according to previous publications.^22,32,64^ Toluidine blue staining was performed and quantified for osteoblast parameters (N.Ob/BS, number of osteoblasts over bone surface; Ob.S/BS, osteoblast surface over bone surface), as previously established.^23,65,66^ TRAP staining was performed and quantified for osteoclast/bone resorption examination (N.Oc/BS, number of osteoclasts over bone surface; Oc.S/BS, osteoclast surface over bone surface), as stated before.^22,23,32^

### IHC and IF staining

According to previous studies,^24,65^ femora were fixed, decalcified and embedded in paraffin for IHC staining, or sucrose-cryoprotected and embedded in the optimal cutting temperature compound for IF staining. Proximal metaphyses were sectioned and underwent IHC staining with a rabbit anti-mouse Pdcd4 primary antibody (9535, Cell Signaling Technology, USA), or underwent IF staining with either a rabbit anti-mouse F4/80 primary antibody (ab111101, Abcam, UK) or a mouse anti-mouse Cd11c primary antibody (53-0114-82, Invitrogen, USA), accordingly.^24,65^ Positively stained cells/area over total area was quantified using the ImageJ 1.47 software.

### Statistical analysis

Data are represented as the mean ± standard deviation. Statistical significance was evaluated by two-tailed Student’s *t* test for two-group comparison, or by one-way analysis of variation followed by Newman-Keuls post hoc tests for multiple comparisons using the Prism 5.01 software (GraphPad, USA). Values of *P* < 0.05 were considered statistically significant.

## Supplementary information

Supporting information

## Data Availability

The data are available from the corresponding authors upon reasonable request.
