# Supplementary material for: Targeted inhibition of osteoclastogenesis reveals the pathogenesis and therapeutics of bone loss under sympathetic neurostress
Source: Int J Oral Sci. 2022 Aug 1;14:39. doi: 10.1038/s41368-022-00193-1 (PMC9343357; doi:10.1038/s41368-022-00193-1)
Supplement: Supplementary file 1 — Supporting information [file 41368_2022_193_MOESM1_ESM.pdf]

Supporting Information

Targeted Inhibition of Osteoclastogenesis Reveals the Pathogenesis and Therapeutics of Bone Loss under Sympathetic Neurostress

Running Title: Sympathetic Control of Bone *via* Osteoclast MiR-21

Bing-Dong Sui[1,#], Jin Liu[2,3,#], Chen-Xi Zheng[1], Lei Dang[2], Ji Chen[1], Yuan Cao[1], Kai-Chao Zhang[1,4], Lu Liu[1], Min-Yan Dang[1,4], Li-Qiang Zhang[1,4], Nan Chen[1], Tao He[1], Kun Xuan[1], Fang Jin[1], Ge Zhang[2], Yan Jin[1,4,*], and Cheng-Hu Hu[1,3,4,5,*]

[1] State Key Laboratory of Military Stomatology & National Clinical Research Center for Oral Diseases & Shaanxi International Joint Research Center for Oral Diseases, Center for Tissue Engineering, School of Stomatology, The Fourth Military Medical University, Xi’an, Shaanxi 710032, China.

[2] Law Sau Fai Institute for Advancing Translational Medicine in Bone & Joint Diseases, School of Chinese Medicine, Hong Kong Baptist University, Hong Kong SAR, China.

[3] Xi’an Key Laboratory of Stem Cell and Regenerative Medicine, Institute of Medical Research, Northwestern Polytechnical University, Xi’an, Shaanxi 710072, China.

[4] Xi'an Institute of Tissue Engineering and Regenerative Medicine, Xi'an, Shaanxi 710032, China.

[5] Department of Biochemistry and Molecular Biology, School of Basic Medical Sciences, Xi'an Jiaotong University, Xi'an, Shaanxi 710032, China.

[#] These authors contributed equally to this study.

* Corresponding authors: Dr. Cheng-Hu Hu and Prof. Yan Jin

Supplementary Figures and Legends

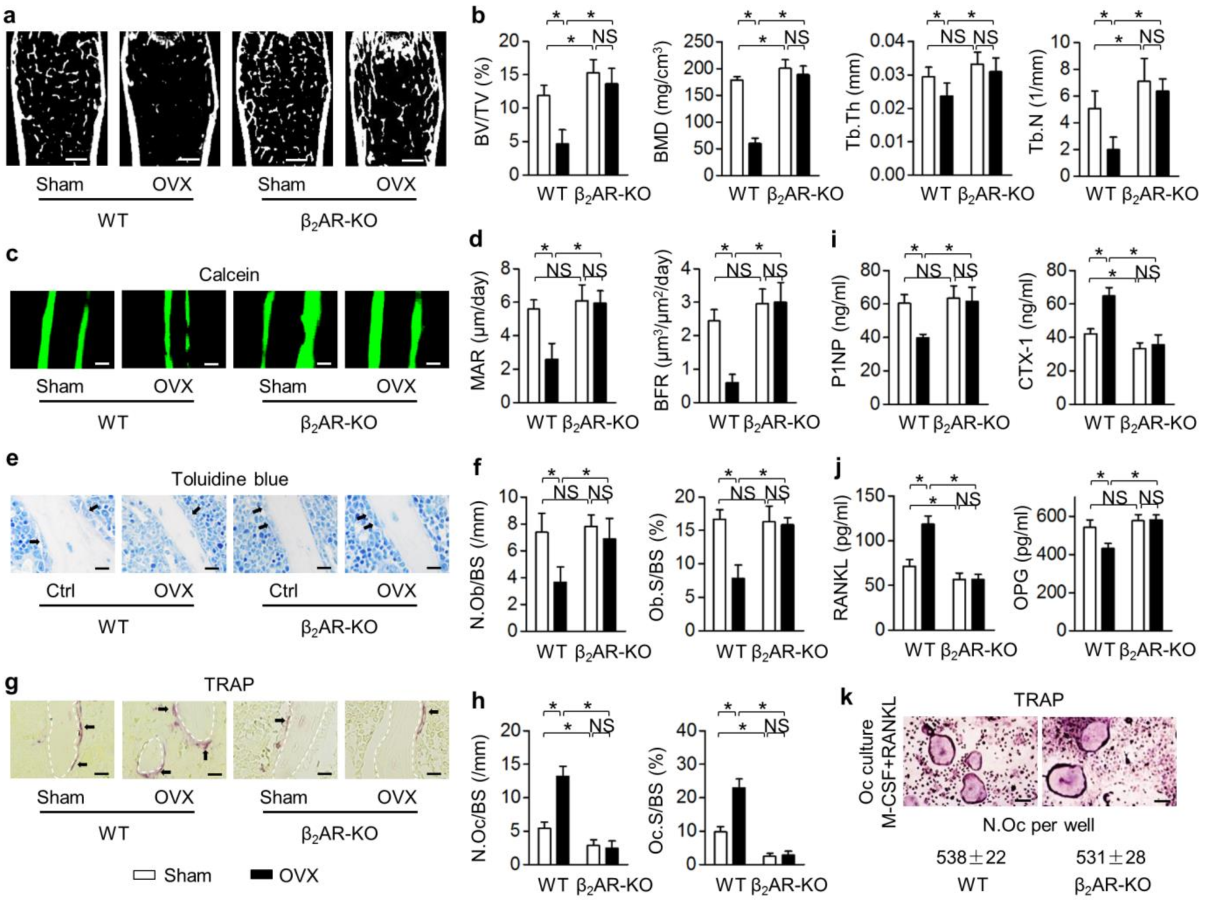

Figure S1. Genetic deficiency of β$_2$AR preserves bone homeostasis against the detrimental effects of OVX. (**a, b**) Micro-CT analysis of femoral bone mass and trabecular parameters. Bars: 500 μm. (**c, d**) Calcein labeling of mineralized femoral surface and quantification of bone formation rates. Bars: 50 μm. (**e, f**) Toluidine blue staining showing osteoblasts (black arrows indicated) and the quantification. Bars: 12.5 μm. (**g, h**) TRAP staining for osteoclasts (black arrows indicated) and the quantification. White dashed lines indicate borders of the trabecular bone. Bars: 12.5 μm. (**i, j**) ELISA analysis of serum bone remodeling markers. (**k**) TRAP staining shows osteoclasts (Oc) formed by M-CSF and RANKL stimulation. N = 6 per group (**a-j**) and N = 4 per group (**k**). WT or β$_2$AR knock-out mice received Sham or OVX surgery. Mean ± SD. *, P < 0.05; NS, not significant.

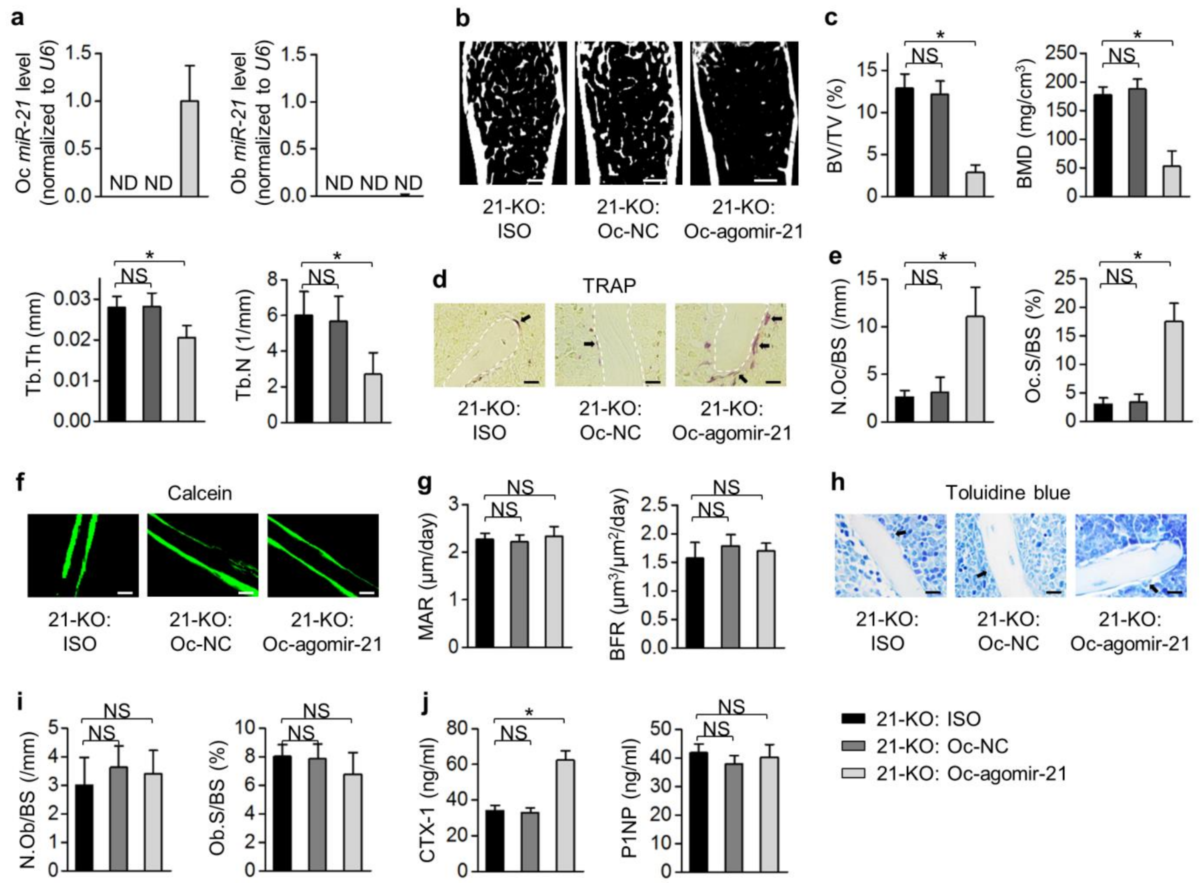

Figure S2. Osteoclastic miR-21 *per se* is critical for osteoclastogenesis and ISO-induced bone loss. (**a**) MiR-21 expression levels in isolated osteoclasts (Oc) and osteoblasts (Ob). (**b, c**) Micro-CT analysis of femoral bone mass and trabecular parameters. Bars: 500 μm. (**d, e**) TRAP staining for osteoclasts (black arrows indicated) and the quantification. White dashed lines indicate borders of the trabecular bone. Bars: 12.5 μm. (**f, g**) Calcein labeling of mineralized femoral surface and quantification of bone formation rates. Bars: 50 μm. (**h, i**) Toluidine blue staining showing osteoblasts (black arrows indicated) and the quantification. Bars: 12.5 μm. (**j**) ELISA analysis of serum bone remodeling markers. $N = 3$ per group (**a**) and $N = 5$ per group (**b-j**). Osteoclast-targeted delivery of agomir-21 (Oc-agomir-21) or its negative control (Oc-NC) was performed in miR-21 knock-out mice under the ISO challenge. Mean ± SD. *, $P < 0.05$; NS, not significant.

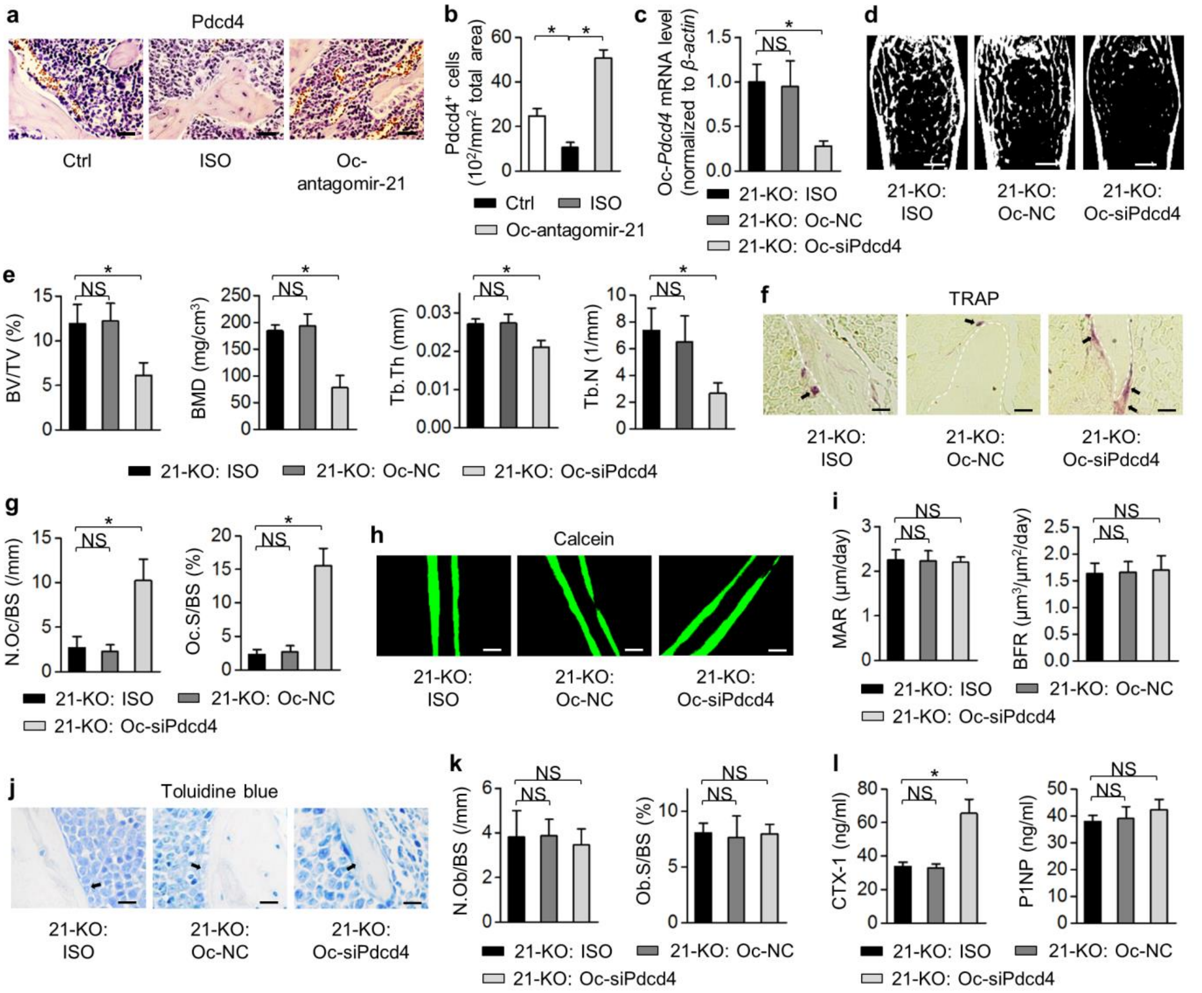

Figure S3. Pdcd4 is the target of miR-21 in response to ISO and regulate osteoclastogenesis. (**a, b**) IHC staining and quantification of Pdcd4 in mouse distal femora. Osteoclast-targeted delivery of antagomir-21 (Oc-antagomir-21) was performed under the ISO challenge. Bars: 100 μm. (**c**) Pdcd4 expression levels in isolated osteoclasts (Oc). (**d, e**) Micro-CT analysis of femoral bone mass and trabecular parameters. Bars: 500 μm. (**f, g**) TRAP staining for osteoclasts (black arrows indicated) and the quantification. White dashed lines indicate borders of the trabecular bone. Bars: 12.5 μm. (**h, i**) Calcein labeling of mineralized femoral surface and quantification of bone formation rates. Bars: 50 μm. (**j, k**) Toluidine blue staining showing osteoblasts (black arrows indicated) and the quantification. Bars: 12.5 μm. (**l**) ELISA analysis of serum bone remodeling markers. *N* = 6 per group (**a, b**), *N* = 3 per group (**c**) and *N* = 5 per group (**d-l**). Osteoclast-targeted delivery of siRNA for Pdcd4 (Oc-siPdcd4) or its negative control (Oc-NC) was performed in miR-21 knock-out mice under the ISO challenge (**c-l**). Mean ± SD. *, $P < 0.05$; NS, not significant.
